# Draft sequencing and assembly of the genome of the world’s largest fish, the whale shark: *Rhincodon typus* Smith 1828

**DOI:** 10.1186/s12864-017-3926-9

**Published:** 2017-07-14

**Authors:** Timothy D. Read, Robert A. Petit, Sandeep J. Joseph, Md. Tauqeer Alam, M. Ryan Weil, Maida Ahmad, Ravila Bhimani, Jocelyn S. Vuong, Chad P. Haase, D. Harry Webb, Milton Tan, Alistair D. M. Dove

**Affiliations:** 1 Department of Medicine, Division of Infectious Diseases, Emory University School of Medicine, 1760 Haygood Drive, Atlanta, GA 30322 USA; 2 Department of Human Genetics, Emory University School of Medicine, 1760 Haygood Drive, Atlanta, GA 30322 USA; 3 Georgia Aquarium, 225 Baker Street, Atlanta, GA 30313 USA

**Keywords:** Fish, Whole genome shotgun, Whale shark, *Rhincodon typus*, Elasmobranch, Gnathostomata, Vertebrate

## Abstract

**Background:**

The whale shark (*Rhincodon typus*) has by far the largest body size of any elasmobranch (shark or ray) species. Therefore, it is also the largest extant species of the paraphyletic assemblage commonly referred to as fishes. As both a phenotypic extreme and a member of the group Chondrichthyes – the sister group to the remaining gnathostomes, which includes all tetrapods and therefore also humans – its genome is of substantial comparative interest. Whale sharks are also listed as an endangered species on the International Union for Conservation of Nature’s Red List of threatened species and are of growing popularity as both a target of ecotourism and as a charismatic conservation ambassador for the pelagic ecosystem. A genome map for this species would aid in defining effective conservation units and understanding global population structure.

**Results:**

We characterised the nuclear genome of the whale shark using next generation sequencing (454, Illumina) and de novo assembly and annotation methods, based on material collected from the Georgia Aquarium. The data set consisted of 878,654,233 reads, which yielded a draft assembly of 1,213,200 contigs and 997,976 scaffolds. The estimated genome size was 3.44Gb. As expected, the proteome of the whale shark was most closely related to the only other complete genome of a cartilaginous fish, the holocephalan elephant shark. The whale shark contained a novel Toll-like-receptor (TLR) protein with sequence similarity to both the TLR4 and TLR13 proteins of mammals and TLR21 of teleosts. The data are publicly available on GenBank, FigShare, and from the NCBI Short Read Archive under accession number SRP044374.

**Conclusions:**

This represents the first shotgun elasmobranch genome and will aid studies of molecular systematics, biogeography, genetic differentiation, and conservation genetics in this and other shark species, as well as providing comparative data for studies of evolutionary biology and immunology across the jawed vertebrate lineages.

## Background

**Fig. 1:**
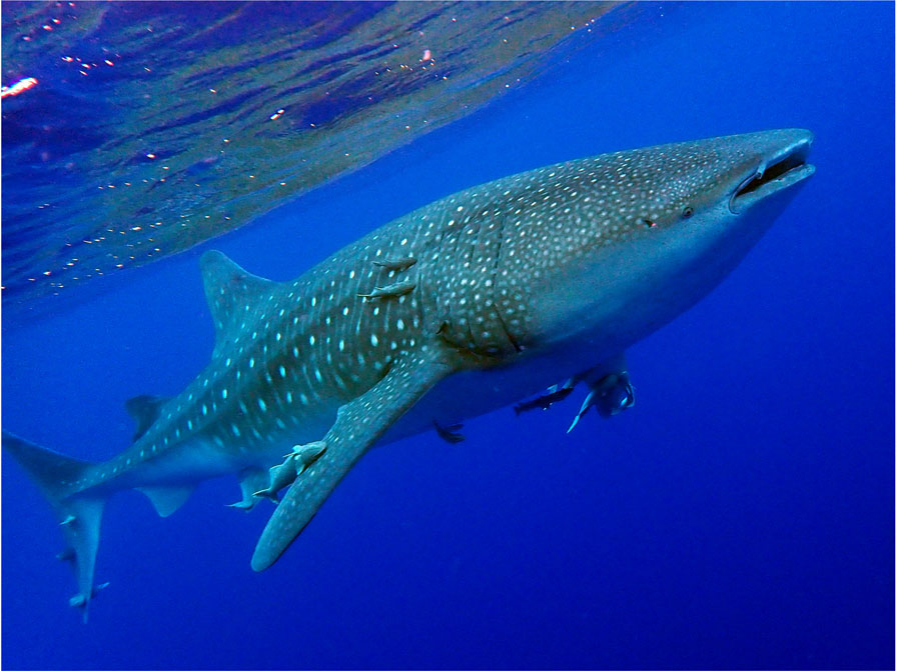
Whale shark (*Rhincodon typus*) from St. Helena (Photo credit: Alistair D.M. Dove. Rights free use permitted)

Until relatively recently, little was known about the biology of the largest shark in the world, the circum-tropical, filter-feeding whale shark, *Rhincodon typus* Smith 1828 [1–4] (Fig. 1). Advances in tagging technology, combined with the discovery of several reliable, seasonal, near-coastal aggregations in different parts of the world [3, 5, 6] have spurred a rapid expansion in whale shark science since 2000. These efforts have been further enhanced by the three International Whale Shark Conferences (the most recent collected at [7]), which have served to promote collaboration on what is otherwise a fairly intractable species to study, due to its size and oceanic habits. The maintenance of a collection of whale sharks at Georgia Aquarium has provided research opportunities not previously available in the natural setting of whale sharks, including the ability to collect samples suitable for genome sequencing. *R. typus* is an excellent model for comparative genomic study because cartilaginous fishes form the sister group to the remaining gnathostomes, because it represents a phenotypic extreme in body size among sharks and fishes generally, and because it is a charismatic subject of ecotourism, yet globally vulnerable to extinction.

The biology of the whale shark has been previously reviewed [1–4]. The whale shark was first described by Smith in 1828 based on a specimen from South Africa [8]. By far the largest species of fish, the largest confirmed size of a whale shark is 18.8 m in total length [9]. The whale shark is a pelagic filter-feeder and the only member of the family Rhincodontidae, whereas other members of the order Orectolobiformes – to which the species belongs – are benthic, bottom-feeding sharks. The whale shark also has the highest fecundity of any shark, with a single individual found bearing over three hundred developing embryos [10]. This species is primarily found in warm oceanic waters, though it is capable of diving to depths where waters approach freezing temperatures [11]. The whale shark is listed on the International Union for Conservation of Nature’s Red List of threatened species as endangered [12], and is a flagship species in marine conservation. Though the whale shark is targeted by fisheries in several countries and is occasionally taken as bycatch, much of the exploitation of whale sharks is for ecotourism around the world, rather than as a food source [3].

There are few publications on the genetics and genomics of whale sharks. Some of the first efforts at discriminating substructure in the global population were based on microsatellite [13] or mitochondrial control loop [14] sequences and failed to detect as much global population structure as might be expected. In a recent review incorporating natural history data, Sequeira et al. [15] concluded that whale sharks are part of a single global metapopulation. These studies have been contradicted by a more recent paper that found distinct genetic differences between Atlantic and Indo-Pacific whale sharks [16] based on additional microsatellite loci. Alam et al. [17] provided the first genomic exploration of the whale shark: the complete mitochondrial genome along with a phylogenomic comparison with representative members of the other major elasmobranch orders. The number of chromosomes in the whale shark genome has not yet been ascertained.

*Rhincodon typus* and other cartilaginous fishes are members of the Gnathostomata, or jawed vertebrates, a group which arose roughly halfway through the Palaeozoic era, and radiated to produce many of the groups of animals most familiar to the general public: sharks, bony fishes, amphibians, reptiles, birds and mammals, including humans. The transition from jawless to jawed vertebrates included several important adaptations that have defined the success of vertebrate life, including the adoption of antibody-based immune systems [18]. The closest relatives to the gnathostomes are jawless fishes, represented among extant taxa only by hagfish and lamprey [19]. Extant gnathostomes themselves are divided into two major clades: Chondrichthyes (cartilaginous fishes) and Osteichthyes (bony fishes and tetrapods). Cartilaginous fishes consist of holocephalans (ratfishes), and the elasmobranchs (sharks and rays). Comparative studies including cartilaginous fishes thus can provide insight into the origin and evolution of jawed vertebrates. Furthermore, cartilaginous fishes can be important model species for comparative studies of human evolution, including anatomy, physiology and immunology. Venkatesh and co-authors [18, 20–23] have explored the genomic basis of some of these adaptations in the elephant shark, *Callorhinchus milii*, a cartilaginous fish from the Holocephali; however, no elasmobranch species has had a complete nuclear genome published prior to this study. Mitochondrial phylogenetic analysis of the individual sequenced in this study was previously published [17]. Results recapitulated previously known relationships for the whale shark as a member of the order Orectolobiformes, with all five orectolobiform shark species forming a clade. This was congruent with a prior study with higher taxon sampling and fewer mitochondrial genes that placed the whale shark among orectolobiform sharks as the sister group to a clade formed by two species that represented the families Ginglystomatidae and Stegostomatidae [24].

In this short report we present the preliminary whole genome shotgun sequencing analysis of a *R. typus* male. The current data set has already been of use to researchers studying shark biology and the evolution of Gnathostomata. In future work, we will present a more complete genome assembly, which is currently in progress.

## Methods

### Genome project history

The genome sequence was derived from tissue samples opportunistically collected in 2007 postmortem from a male whale shark of Taiwanese origin at Georgia Aquarium, prior to the start of the present study. Samples from this specimen have also been used in studying whale shark brain anatomy [25]. The animal was originally collected near Hualien, Taiwan (23.9722° N, 121.6064° E) in 2004 as part of a pelagic trap fishery quota, and exported with appropriate permission provided by Taiwan. Other details about the genome’s project history and sequencing are summarized in Table 1. Raw data from the project is available from the NCBI short read archive under accession number SRP044374.

**Table 1:** Project information

Property	Term
Finishing quality	High quality draft
Libraries used	Illumina: paired end library; 454: single end library
Sequencing platforms	Illumina HiSeq 2000/454 GS FLX Titanium
Fold coverage	30×
Assemblers	SOAPdenovo (v. 2.04)
Gene calling method	AUGUSTUS. Proteins matched against the NCBI nr database using BLASTP, and the INTERPRO profile database using InterProScan
Genbank ID	LVEK00000000
GenBank Date of Release	5.11.2016
GOLD ID	Gp0102394
BIOPROJECT	PRJNA255419

### Genome sequencing and assembly

The genomic DNA used for this study was isolated from liver and spleen tissues using the Qiagen Maxi Prep kit (Qiagen, Venlo, Netherlands). Purity was assessed using Nanodrop and Agilent Bioanalyzer.

Sequencing was performed using 454 and Illumina technologies at Emory University, HudsonAlpha Instititute, and 454 Inc. Sequencing runs and libraries are summarized in Table 2. After low quality reads were filtered out using *preqc* tool (v. 0.10.13) [26], the remaining reads were assembled using SOAPdenovo (v. 2.04). Assemblies were created using k-mers 31–89 for the de Bruijn graph building step of the algorithm. Statistics for each assembly were generated using a script from the Assemblathon project. K-mer 63 was chosen as the best assembly because this assembly had (a) the largest contig (86,048 bp) and (b) a NG50 very similar to the other top scores (63-mer: 3358 bp, 65-mer: 3454 bp, and 67-mer: 3406 bp). For the final version (called v1, available on GenBank: LVEK00000000), we excluded contigs below 200 bp for downstream analysis.

**Table 2:** Sequencing runs and libraries generated. *Types are SE – single end, PE – paired end, and MP – mate pair

SRA ID	Tissue	Library ID	Technology	Type*	Ave insert size (std dev)	Sequence length (bp)	Number of reads	Total bp
SRR1521182	Spleen	1	LS454	SE	na	401,304	1,268,373	728,329,555
SRR1521184	Spleen	1	LS454	SE	na	401,328	1,279,760	680,625,037
SRR1521184	Spleen	1	LS454	SE	na	401,328	1,279,760	680,625,037
SRR1521191	Spleen	2	Illumina	PE	293(101)	100	210,821,824	21,082,182,400
SRR1521192	Spleen	2	Illumina	PE	300(91)	100	585,821,484	58,582,148,400
SRR1521195	Spleen	2	Illumina	PE	328(90)	100	585,054,464	58,505,446,400
SRR1521197	Spleen	2	Illumina	PE	286(100)	100	224,670,734	22,467,073,400
SRR1521198	Spleen	3	Illumina	MP	7161(755)	100	571,738,680	57,173,868,000
SRR1521199	Spleen	2	Illumina	PE	290(100)	100	300,519,032	30,051,903,200
SRR1521200	Spleen	4	Illumina	SE	na	51	108,403,623	5,420,181,150
SRR1521201	Spleen	5	Illumina	PE	274(54)	100	34,239,020	3,423,902,000
SRR1521204	Spleen	5	Illumina	PE	236(46)	100	90,708,094	9,070,809,400
SRR1521190	Liver	6	Illumina	PE	215(43)	100	99,078,844	9,907,874,400

### Genome annotation

Whale shark proteins were predicted de novo on the assembled contigs using AUGUSTUS (v. 3.0.3)[27]. The proteins were matched against the NCBI nr database using BLASTP (v. 2.2.29+)[28] with a threshold cutoff E-value of 10^−3^, and KronaTools (v2.4) [29] was used to create taxonomic visualizations of these results. Proteins were annotated using the INTERPRO profile database using InterProScan (v5) [30]. COG (core ortholog group) annotations were also annotated using BLASTP (v. 2.2.29+) against the KOG database (the COG database for eukaryotes [31, 32]) with a threshold cutoff E-value of 10^−5^.

### Ortholog analysis

In order to investigate ortholog patterns we compared the predicted *R. typus* proteome against proteomes from 10 other fishes and lamprey using BLASTP with a cutoff E-value of 10^−5^ and clustered into groups related by sequence similarity with the ORTHOMCL software pipeline. The predicted proteomes of Atlantic cod (*Gadus morhua*, accession GCA_000231765.1) [33], coelacanth (*Latimeria chalumnae*, GCA_000225785.1) [34], fugu (*Takifugu rubripes*, GCA_000180615.2) [35], elephant shark (*Callorhinchus milii*, GCA_000165045.2*)* [18]*,* sea lamprey (*Petromyzon marinus*, GCA_000148955.1) [36], medaka (*Oryzias latipes*, Ensembl MEDAKA1) [37], Nile tilapia (*Oreochromis niloticus*, GCA_000188235.1) [38], stickleback (*Gasterosteus aculeatus*, GCA_000180675.1) [39], green spotted pufferfish (*Tetraodon nigroviridis*, GCA_000180735.1) [40], and zebrafish (*Danio rerio*, GCA_000002035.3) [41] were downloaded from the UCSC genome browser site [42] in November 2014. The annotated complete predicted proteomes were combined into a single database and searched against itself (all vs all) using BLASTP (v.2.2.30) with a threshold cutoff E-value of 10^−5^. The percent identity, E-value and alignment scores were parsed out from the BLASTP output in order to compute the percent match identity, which were utilized for identifying the orthologous sequences using the OrthoMCL algorithm [43]. Core genes are defined as the protein-coding gene clusters that are shared by all fish genomes used in this study. Unique genes found in only one of the fish genomes were also identified in this analysis. MUSCLE (v. 3.6) [44] was used with default settings to align the core genes, and each of the protein alignments was filtered by GBLOCKS (v0.91) [45] to remove gaps and highly divergent regions. Core gene sequences were concatenated for phylogenomic analysis. Maximum likelihood (ML) phylogenetic reconstruction was implemented using RAxML (v 7.2.8-ALPHA) [46]. The Jones-Taylor-Thornton (JTT) amino acid substitution model[47] of rate heterogeneity with 4 discrete rate categories was used. To evaluate statistical support, 100 bootstrap replicates were computed. Zebrafish proteins with orthologs missing in the whale shark were tested for functional significance using WebGestalt (update 5/20/2014) [48].

## Results and discussion

### Genome assembly statistics

Genome assembly statistics are summarized in Table 3. Reference-free analysis of the quality filtered data using the *preqc* tool (v. 0.10.13) [26] gave us an estimate of the genome size based on k-mer word frequency of 3.44 Gb, within the range reported size of other chondrichthyans [49, 50]. The assembly consisted of 1,213,000 contigs and 997,976 scaffolds, a contig N50 of 5304 bp, and a scaffold N50 of 5425 bp. We estimated that we had approximately 30-fold redundancy in coverage of the genome. The DNA composition of the assembled contigs was 41.3% G + C. The rather low N50 compared to other recent vertebrate genome projects suggests that the assembly could benefit from more mate-pair and long read sequences, as well as deeper coverage of Illumina sequence to help correct sequence. The assembly incorporated an Illumina mate-pair library of approximately 3 kb. Attempts to construct larger insert mate-pair libraries resulted in failure.

**Table 3:** Genome and predicted protein statistics. Percentages of total genome size calculated as proportion of assembly size rather than estimated genome size

Attribute	Value	% of Total
Genome size (Gbp)	3.44	
DNA coding (bp)	10,400,226	0.41%
DNA G + C (bp)	1,059,229,091	41.3%
Number of scaffolds	997,976	
Scaffold N50 (bp)	5425	
Number of contigs	1,213,000	
Contig N50 (bp)	5304	
Protein coding genes	19,384	
Genes with function prediction	5380	27.8%
Genes assigned to KOGs	7038	36.3%
Genes with Pfam domains	6612	34.1%

Sequence contamination is an issue that has bedeviled whole-genome sequencing projects [51]. We therefore expected to see non-whale shark DNA originating from carryover from previous Illumina runs, and contamination from extrinsic laboratory sources during tissue preparation, the latter especially since the *R. typus* diet may contain unusually high levels of bacteria [52]. To determine the approximate extent of contamination, we used BLAST to compare the assembly to the highly conserved bacterial 16S gene and found only four contigs with low sequence coverage (5–7 fold redundancy) had greater than 75% matches to the whole gene. Therefore, we concluded that bacterial contamination was present but not a major factor in this project.

Immediately prior to the public release of these data (December 2014) there were only 110 nucleotide sequences in the NCBI database assigned a *R. typus* taxonomic origin. 109/110 of these sequences could be mapped to the contigs from this project with a threshold match significance BLAST score of 10^−5^ or lower. The one sequence that did not match was a putative recombination activating protein 2 ortholog (NCBI gid:315,571,864) that turned out to have best matches only to other bony fishes and thus may have been misidentified in its origin.

### Predicted proteins

**Fig. 2:**
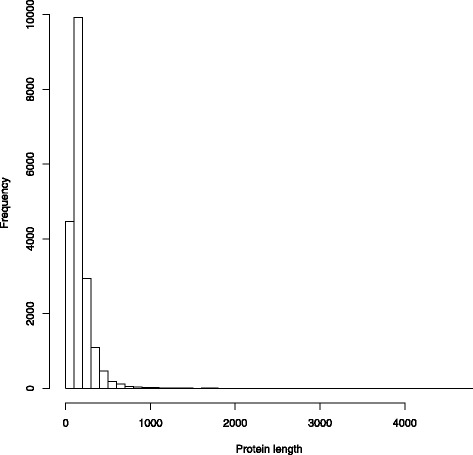
Histogram of predicted protein sizes

**Fig. 3:**
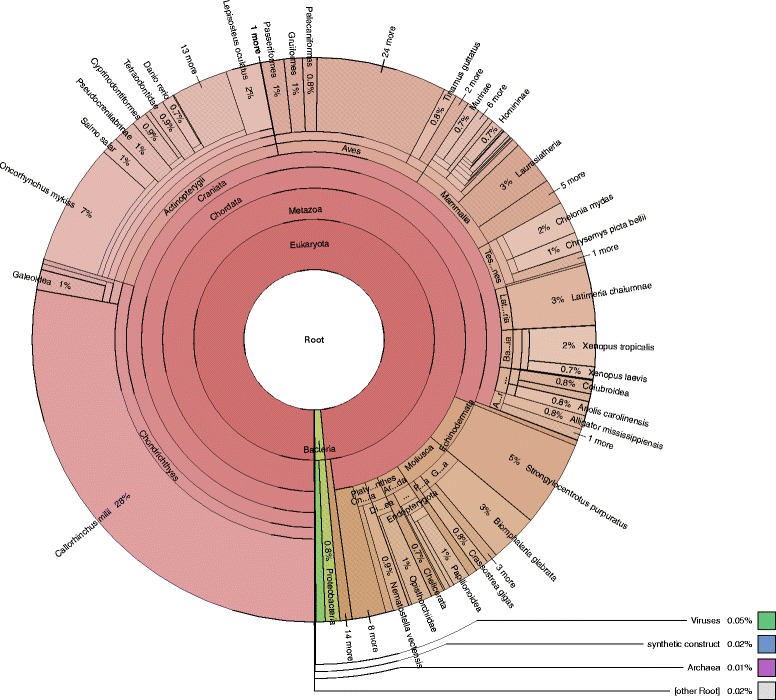
Overview of best matches to the protein database that map to the Chordata taxonomy group

**Fig. 4:**
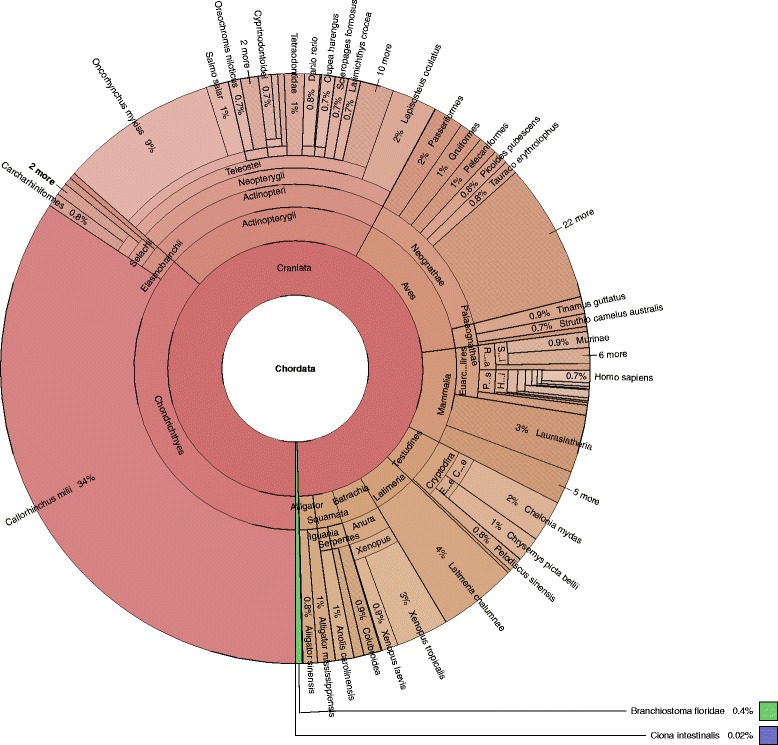
Phylogeny based on alignment of conserved single-copy proteins. Silhouettes are not to scale. Accessions: *Petromyzon*: GCA_000148955.1, *Callorhinchus*: GCA_000165045.2, *Latimeria*: GCA_000225785.1, *Danio*: GCA_000002035.3, *Gadus*: GCA_000231765.1, *Gasterosteus*: GCA_000180675.1, *Oryzias*: version MEDAKA1 (Ensembl), *Oreochromis*: GCA_000188235.1, *Takifugu*: GCA_000180615.2, *Tetraodon*: GCA_000180735.1. Silhouette credits: *Petromyzon* by Gareth Monger, CC-BY; *Callorhinchus* by Tony Ayling, CC-BY-SA; *Rhincodon* by Scarlet23, vectorized by T. Michael Keesey, CC-BY-SA; *Latimeria* by Maija Karala, CC-BY-NC-SA; *Gadus*, *Oreochromis*, *Tetraodon*, *Gasterosteus* by Milton Tan; *Danio*, *Oryzias*, *Takifugu*, no copyright

Use of the AUGUSTUS software [27] for de novo gene prediction resulted in 19,384 protein-coding genes predicted on the assembled contigs (available on Figshare [53]). While the largest predicted protein was 4709 amino acids in length, the majority of the proteins were less than 200 amino acids (Fig. 2). Of the predicted proteins, 14,736 (76%) of the proteins had a blastp match in the NCBI nr database. More than 99% of the protein best matches were to eukaryotes (Fig. 3), providing further evidence that prokaryotic contamination in the project was limited. Within the eukaryotes, 82% of the matches were to Chordata, with other fish species that have completed genomes as predominant matches (Fig. 4). The genome with the greater number of best matches (34% of Chordata) was the elephant shark. These results were therefore in line with what would be expected of a novel chondrichthyan genome sequence. Of the predicted proteins, 7038 (36.3%) of the proteins had a blastp match in the KOG database (Table 4).

**Table 4:** Number of genes associated with general KOG functional categories. Percentages of genes is based on the total number of predicted proteins

Code	Value	%	Description
J	161	0.83	Translation, ribosomal structure and biogenesis
A	226	1.17	RNA processing and modification
K	458	2.36	Transcription
L	128	0.66	Replication, recombination and repair
B	154	0.79	Chromatin structure and dynamics
D	143	0.74	Cell cycle control, Cell division, chromosome partitioning
V	100	0.52	Defense mechanisms
T	1280	6.60	Signal transduction mechanisms
M	52	0.27	Cell wall/membrane biogenesis
N	22	0.11	Cell motility
U	307	1.58	Intracellular trafficking and secretion
O	532	2.74	Posttranslational modification, protein turnover, chaperones
C	105	0.54	Energy production and conversion
G	165	0.85	Carbohydrate transport and metabolism
E	140	0.72	Amino acid transport and metabolism
F	65	0.34	Nucleotide transport and metabolism
H	19	0.10	Coenzyme transport and metabolism
I	164	0.85	Lipid transport and metabolism
P	345	1.78	Inorganic ion transport and metabolism
Q	66	0.34	Secondary metabolites biosynthesis, transport and catabolism
R	2142	11.06	General function prediction only
S	393	2.03	Function unknown
-	-	-	Not in KOGs

The total is based on the total number of protein coding genes in the genome

### Ortholog analysis

**Fig. 5:**
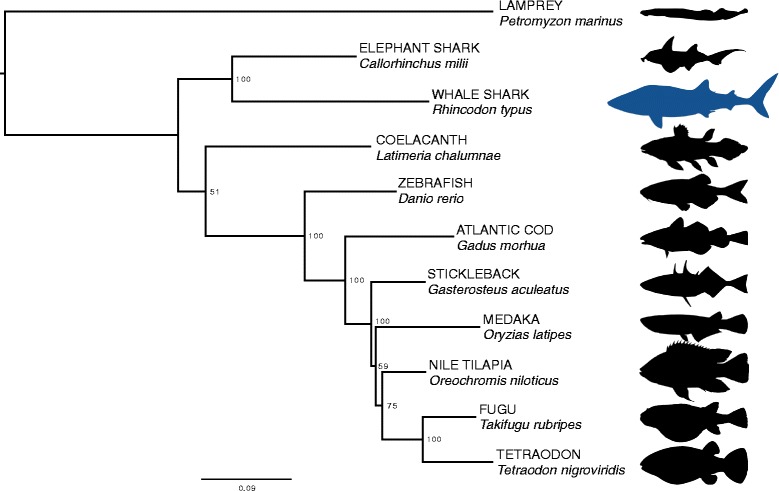
Overview of taxonomy of whale shark protein best matches to the nr database. Figure was constructed from best BLAST matches to the nr database using Krona [31] tool

From comparisons of the whale shark genome with ten other fish genomes, we found that there was a ‘core’ set of 1846 ortholog groups with at least one protein member present in each of the eleven genomes, representing a set of highly conserved functions. Of these genes, 155 orthologs were present with exactly one protein member in each of the groups. The phylogeny based on concatenation of these core genes recapitulated the established evolutionary relationship of the species: the cartilaginous fishes *R. typus* and *C. milii* form a deep clade as the sister clade to bony fishes (Fig. 5).

The ortholog analysis revealed that there were 865 protein families present in the other genomes that were missing in the whale shark. This number was of the same order as the outgroup lamprey genome (764 missing orthologs) and higher than that seen in the other fishes (the elephant shark genome had only 108 missing protein sequences). Further, there were 543 proteins missing from both the whale shark and lamprey but represented in all the other nine genomes. These absent proteins could be explained by some combination of the draft nature of the sequence data in this project, the preliminary de novo annotation, or the evolutionary divergence of the whale shark and lamprey from the other species. We mapped the orthologs of the missing proteins in the well-annotated zebrafish genome and tested for enrichment of terms in the Gene Ontology or Kyoto Encyclopedia of Genes and Genomes databases using the WebGestalt GSAT analysis tool [48]. We found no specifically enriched terms or pathways in the missing protein set compared to the entire zebrafish proteome. This suggested that the absent genes were not overrepresented in any particular functional category, as might have happened through adaptive gene deletion.

The remaining 4648 predicted proteins with no nr database match tended to be short (mean of 126.5 amino acids, compared to 179 for the protein dataset as a whole), suggesting that many were annotation overcalls, or fragments of proteins disrupted by contig gaps. Several of these proteins are large enough that they are unlikely to be the result of spurious translation (9 were >500 amino acids in length, the largest 1352 amino acids). These could represent novel chondrichthyan genes, although it is also possible that many of the proteins without a best match could be uncultivated microorganisms.

### Preliminary comparisons

The only other cartilaginous fish for which a complete genome has been assembled is the elephant shark *C. milii* [18, 20, 23], which is not an elasmobranch but a member of the Holocephali (also known as ratfishes). There are striking differences between the genomes, most obviously in size. The whale shark genome, at 3.44 Gb, is approximately 3.5× the size of the elephant shark genome at only 950 Mb. The genomes were also diverged at the DNA level. In a discontiguous megablast alignment between the *C. milii* and whale shark scaffolds, the combined length of matches with an E value of <0.001 was only 42 Mb of the elephant shark genome (71% nucleotide identity). In addition, based on our phylogenetic analysis, the number of estimated substitutions is higher in whale shark than in elephant shark.

Comparisons of cartilaginous fishes such as *C. milii* and *R. typus* to other vertebrates can provide some insight into the evolution of jawed vertebrates. Some of the features of the protein set of *R. typus* recapitulated discoveries made in *C. milii*. For example, homologs of the human SCP and SIBLING proline-glutamine families of bone-deposition proteins were missing from the whale shark genome based on negative results of BLASTX alignment against the scaffolds, a result also seen in the other cartilaginous fishes [18]. *C. milii* is reported to have a pseudogenized copy of the important innate immunity protein Toll-like receptor 4 (TLR4), which detects lipopolysaccharide of infecting Gram negative bacteria [18]. We found that the human TLR4 protein had a significant match (BLASTP 1e^-45^) to a 925 residue protein containing multiple leucine-rich repeat domains and a C-terminal TIR domain (Toll/Interleukin receptor) of the nucleotide-binding TLR2 superfamily. BLAST of this sequence to nr found best hits of this TLR protein were to TLR21 and TLR13. Neither TLR13 nor TLR21 have been previously described in chondrichthyans, with representative taxa including amphibians, mammals, birds, and teleosts [54]. TLR13 and TLR21 have been previously found to be similar, and form a clade within the other TLRs [54]. This whale shark TLR may represent an ancient homolog of these TLRs, and demonstrates these TLRs may have originated in the most recent common ancestor of jawed vertebrates. The whale shark genome will be useful for comparative studies of the origins of jawed vertebrate genes, such as these TLRs.

## Conclusions

We pursued a strategy of primarily using cost-effective Illumina short read sequencing to produce a preliminary *R. typus* genomic dataset. This allowed us to maximize coverage of the genome with high quality data and give estimates of the genome size and extent of bacterial contamination of the source DNA (both unknown at the start of the project), and to provide what we believe is a quite complete, if fragmented, draft of the genome. De novo gene prediction and comparisons with other fish genomes suggest the gene content and phylogenetic relationships of the proteins were generally as expected of a cartilaginous fish. Future work will enhance the whale shark genome assembly using long reads using the Pacific Biosciences technology. The genome assembly will also be further enhanced by incorporating RNA-seq data to aid gene annotation, although technical and ethical constraints on obtaining samples from live animals may limit our work to archived tissues.

The genome sequence of an organism is now perhaps the single most important gateway to understanding its biology. We believe that despite the incomplete nature of the data, the draft sequence presented here will be a resource that can accelerate scientific investigation of the whale shark and of elasmobranchs in general. We have shown that the data encompasses almost all the current publicly-submitted whale shark nucleotide sequences. Although, many genes are likely split over two or more contigs, and the large number of putatively ‘missing’ proteins probably reflects this reality in the draft sequence. Some caution should therefore be used when concluding that a protein homolog is missing from these data. Nevertheless, the current DNA sequence can be mined for new genotyping tools for population genomics and the protein set can be compared intensively against known functions. The long term goals include understanding the genetic nature of the large body size of the whale shark, its metabolic adaptations to its planktonic diet, and the evolution of its immune system in a comparative context within the gnathostomes.

This public data set is not only for research but can also be a teaching tool. We used an intramural version of the Galaxy server in a basic bioinformatics analysis course for undergraduates at Emory University (three of whom are on this author list). Students were inspired to improve their bioinformatic skills by the opportunity to explore the vast dataset of this wonderful organism. There are surely many important discoveries that will come from further careful analysis of the genome sequence.

## Abbreviations

COG/KOG: Core ortholog group
JTT: Jones-Taylor-Thornton (amino acid substitution matrix)
ML: maximum likelihood
TLR: Toll-like receptor
