# Erratum to: draft sequencing and assembly of the genome of the world’s largest fish, the whale shark: *Rhincodon typus* smith 1828

**DOI:** 10.1186/s12864-017-4138-z

**Published:** 2017-09-25

**Authors:** Timothy D. Read, Robert A. Petit, Sandeep J. Joseph, Tauqeer Alam, M. Ryan Weil, Maida Ahmad, Ravila Bhimani, Jocelyn S. Vuong, Chad P. Haase, D. Harry Webb, Milton Tan, Alistair D.M. Dove

**Affiliations:** 1 Department of Medicine, Division of Infectious Diseases, Emory University School of Medicine, 1760 Haygood Drive, Atlanta, GA 30322 USA; 2 Department of Human Genetics, Emory University School of Medicine, 1760 Haygood Drive, Atlanta, GA 30322 USA; 3 Georgia Aquarium, 225 Baker Street, Atlanta, GA 30313 USA

## Erratum:

After publication of the original article [1], the authors noted that the following errors had occurred:

1. The following figure captions were in the incorrect order:

- Figure 3: The current caption for Fig. 3 is incorrect and should be the caption for Fig. 4.

**Fig. 3:**
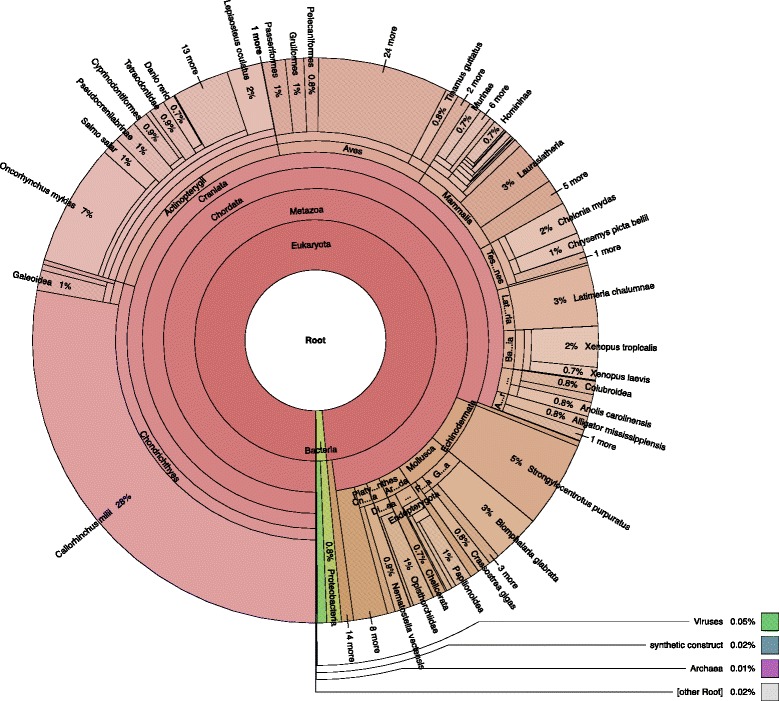
Overview of taxonomy of whale shark protein best matches to the nr database. Figure was constructed from best BLAST matches to the nr database using Krona [31] tool

**Fig. 4:**
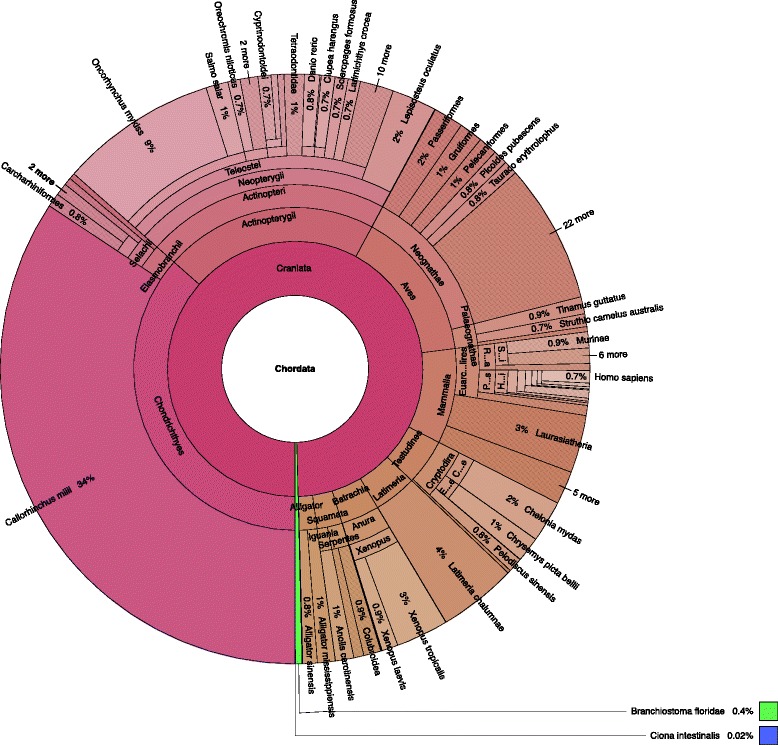
Overview of best matches to the protein database that map to the Chordata taxonomy group

The correct Figure legend should be: **Overview of taxonomy of whale shark protein best matches to the nr database. Figure was constructed from best BLAST matches to the nr database using Krona [31] tool.**

A fully updated Figure including the revised caption is included with this Erratum (Corrected Figure 3).

- Figure 4: The current caption for Fig. 4 is incorrect and should be the caption for Fig. 5.

**Fig. 5:**
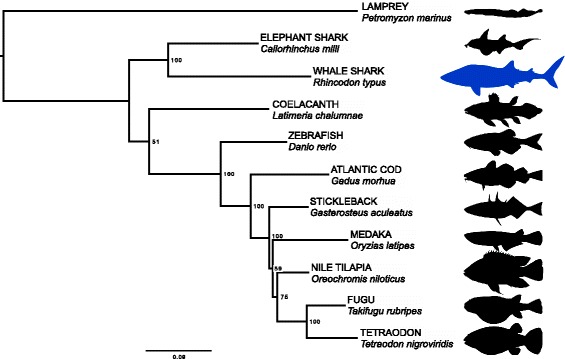
Phylogeny based on alignment of conserved single-copy proteins. Silhouettes are not to scale. Accessions: Petromyzon: GCA_000148955.1, Callorhinchus: GCA_000165045.2, Latimeria: GCA_000225785.1, Danio: GCA_000002035.3, Gadus: GCA_000231765.1, Gasterosteus: GCA_000180675.1, Oryzias: version MEDAKA1 (Ensembl), Oreochromis: GCA_000188235.1, Takifugu: GCA_000180615.2, Tetraodon

The correct Figure legend should be: **Overview of best matches to the protein database that map to the Chordata taxonomy group.**

A fully updated Figure including the revised caption is included with this Erratum (Corrected Figure 4).

- Figure 5: The current caption for Fig. 5 is incorrect and should be the caption for Fig. 3.

The correct Figure legend should be: **Phylogeny based on alignment of conserved single-copy proteins. Silhouettes are not to scale. Accessions: Petromyzon: GCA_000148955.1, Callorhinchus: GCA_000165045.2, Latimeria: GCA_000225785.1, Danio: GCA_000002035.3, Gadus: GCA_000231765.1, Gasterosteus: GCA_000180675.1, Oryzias: version MEDAKA1 (Ensembl), Oreochromis: GCA_000188235.1, Takifugu: GCA_000180615.2, Tetraodon: GCA_000180735.1. Silhouette credits: Petromyzon by Gareth Monger, CC-BY; Callorhinchus by Tony Ayling, CC-BY-SA; Rhincodon by Scarlet23, vectorized by T. Michael Keesey, CC-BY-SA; Latimeria by Maija Karala, CC-BY-NC-SA; Gadus, Oreochromis, Tetraodon, Gasterosteus by Milton Tan; Danio, Oryzias, Takifugu, no copyright.**

A fully updated Figure including the revised caption is included with this Erratum (Corrected Figure 5).

2. All mentions of the GenBank ID LVEK00000000 are incorrect, and should be LVEK00000000.1. This was present in both Table 1 of the original article, and in the Availability of Data and Materials section. In updated version of Table 1 (Corrected Table 1) has been included with this Erratum.

**Table 1:** Project information

Property	Term
Finishing quality	High quality draft
Libraries used	Illumina: paired end library; 454: single end library
Sequencing platforms	Illumina HiSeq 2000/454 GS FLX Titanium
Fold coverage	30×
Assemblers	SOAPdenovo (v. 2.04)
Gene calling method	AUGUSTUS. Proteins matched against the NCBI nr database using BLASTP, and the INTERPRO profile database using InterProScan
Genbank ID	LVEK00000000.1
GenBank Date of Release	5.11.2016
GOLD ID	Gp0102394
BIOPROJECT	PRJNA255419

The original article has also been corrected.

**Corrected Fig.** **3** **:**

**Corrected Fig.** **4** **:**

**Corrected Fig.** **5** **:**

**Corrected Table** **1** **:**
